# Development of a Physiologically Based Pharmacokinetic Model for Hydroxychloroquine and Its Application in Dose Optimization in Specific COVID-19 Patients

**DOI:** 10.3389/fphar.2020.585021

**Published:** 2021-02-12

**Authors:** Miao Zhang, Xueting Yao, Zhe Hou, Xuan Guo, Siqi Tu, Zihan Lei, Zhiheng Yu, Xuanlin Liu, Cheng Cui, Xijing Chen, Ning Shen, Chunli Song, Jie Qiao, Xiaoqiang Xiang, Haiyan Li, Dongyang Liu

**Affiliations:** ^1^ Drug Clinical Trial Center, Peking University Third Hospital, Beijing, China; ^2^ School of Basic Medicine and Clinical Pharmacy, China Pharmaceutical University, Nanjing, China; ^3^ Department of Respiratory, Peking University Third Hospital, Beijing, China; ^4^ Department of Orthopedics, Peking University Third Hospital, Beijing, China; ^5^ Department of Obstetrics and Gynecology, Peking University Third Hospital, Beijing, China; ^6^ Department of Clinical Pharmacy, School of Pharmacy, Fudan University, Shanghai, China; ^7^ Department of Cardiology and Institute of Vascular Medicine, Peking University Third Hospital, Beijing, China

**Keywords:** hydroxychloroquine, physiologically-based pharmacokinetic, drug-drug interaction, specific populations, dosing recommendation

## Abstract

In Feb 2020, we developed a physiologically-based pharmacokinetic (PBPK) model of hydroxychloroquine (HCQ) and integrated *in vitro* anti-viral effect to support dosing design of HCQ in the treatment of COVID-19 patients in China. This, along with emerging research and clinical findings, supported broader uptake of HCQ as a potential treatment for COVID-19 globally at the beginning of the pandemics. Therefore, many COVID-19 patients have been or will be exposed to HCQ, including specific populations with underlying intrinsic and/or extrinsic characteristics that may affect the disposition and drug actions of HCQ. It is critical to update our PBPK model of HCQ with adequate drug absorption and disposition mechanisms to support optimal dosing of HCQ in these specific populations. We conducted relevant *in vitro* and *in vivo* experiments to support HCQ PBPK model update. Different aspects of this model are validated using PK study from 11 published references. With parameterization informed by results from monkeys, a permeability-limited lung model is employed to describe HCQ distribution in the lung tissues. The updated model is applied to optimize HCQ dosing regimens for specific populations, including those taking concomitant medications. In order to meet predefined HCQ exposure target, HCQ dose may need to be reduced in young children, elderly subjects with organ impairment and/or coadministration with a strong CYP2C8/CYP2D6/CYP3A4 inhibitor, and be increased in pregnant women. The updated HCQ PBPK model informed by new metabolism and distribution data can be used to effectively support dosing recommendations for clinical trials in specific COVID-19 patients and treatment of patients with malaria or autoimmune diseases.

## Introduction

Coronavirus disease 2019 (COVID-19) has spread to more than 200 countries around the world since its outbreak in December 2019, and the world gets into a novel coronavirus pandemic. As research in SARS-CoV-2 continues, drugs for the treatment of SARS-CoV-2 infection are emerging, which brings hope for conquering this pandemic. (National Medical Products Administration, 2020) We reported that hydroxychloroquine (HCQ) can inhibit SARS-CoV-2 *in vitro*, and developed physiologically-based pharmacokinetic (PBPK) model incorporating this and other drug disposition information (Yao et al., 2020). We applied this model to support dosing recommendations in two studies in China in a timely manner (ChiCTR2000029898, ChiCTR2000029899). Up to now, the clinical antiviral activity of CQ/HCQ and regulating inflammatory factors of HCQ remain controversial (Ferner and Aronson, 2020; Geleris et al., 2020; Huang et al., 2020; Kox et al., 2020; Meo et al., 2020; Tang et al., 2020; Yu et al., 2020; Zhou et al., 2020) While, HCQ has been recognized regulating inflammatory factors in rheumatoid arthritis whose level of interleukin-6 are fairly with COVID-19 patients (Lee et al., 2017; Kox et al., 2020), so it is deserve to further study the role of HCQ in patients.

Although HCQ has been used clinically for more than 70 years and has been approved for the treatment of malaria, rheumatoid arthritis and lupus erythematosus (Lim et al., 2009), little is known about the mechanisms of its absorption and disposition. US prescribing information of Plaquenil (brand name of hydroxychloroquine sulfate) recommends dose adjustment of HCQ and concomitant medications under different drug combination scenarios, and in specific populations such as subjects with hepatic insufficiency (US Food and Drug Administration, 2019). Hydroxychloroquine sulfate tablets. Since the beginning of the global COVID-19 pandemic, many people have been or are currently exposed to this drug, including specific populations whose underlying intrinsic and/or extrinsic characteristics may affect absorption/disposition and drug efficiency/safety profiles of HCQ. Therefore, it is prudent to develop scientifically sound dose recommendation schemes to facilitate efficient use of HCQ in different populations.

Our earlier HCQ PBPK model (Yao et al., 2020) was effectively applied to support real-time dosing recommendations for our clinicians using HCQ to treat COVID-19 patients in Wuhan. Since then, the model has been used to provide simulations of HCQ in plasma, blood and lung for the development of clinical protocols requested by others. These timely clinical applications highlight the strength of PBPK as an important model-informed drug development approach under emergency situations. However, we also recognize that several limitations of our earlier model may prevent its broader use, especially in specific patient populations. Due to the lack of information, metabolism of HCQ in the liver, a major drug elimination pathway, remain undefined in the model. As such, prediction of the effect of intrinsic and/or extrinsic patient factors (age, pregnancy, organ dysfunction, concomitant medications) on HCQ in specific populations is limited. In addition, a perfusion limited drug distribution mechanism was assumed, and drug accumulation in the lung tissue was achieved by using an additional compartment and changing tissue blood flow rates to mimic the trends of lung-plasma partitioning reported for chloroquine (CQ) in rats (Adelusi and Salako, 1982). The use of fixed partition in the model allows prediction of the steady state drug concentration in the lung tissue, a potential target in COVID-19 patients. However, lung tissue is a blood-rich organ and moderate permeability (P_app_ value of HCQ in Caco-2 Cells is 3.68 × 10^–6^ cm/s, See results *Physiologically-Based Pharmacokinetic Model Development* below) is one of the factors that may restrict HCQ entry into the cells instantaneously. If HCQ gradually enters cells via active process, an updated model is needed to better capture such dynamic drug accumulation inside lung cells.

In this study, we aimed to update our HCQ PBPK model by i) integrating *in vitro* experiments data to further define absorption and metabolism mechanisms of HCQ; ii) performing animal tissue distribution studies and applying permeability-limited model to enable detailed description lung distribution of HCQ, and iii) building the metabolite desethylhydroxychloroquine (DHCQ) PBPK model. The updated model (HCQ-DHCQ PBPK model) therefore has broader capability to support dosing recommendations of HCQ for a variety of specific populations, considering that the severe symptoms of COVID-19 often resulted in liver impairment and occurred in the elderly (Guan et al., 2020; Li and Fan, 2020; Zhou et al., 2020) who usually have renal or hepatic insufficiency. Simulations using the updated PBPK model can also be used to facilitate dosing optimizations for specific populations around the world who may take HCQ for treating malaria or autoimmune diseases.

## Materials and Methods

### Study Strategy

**FIGURE 1:**
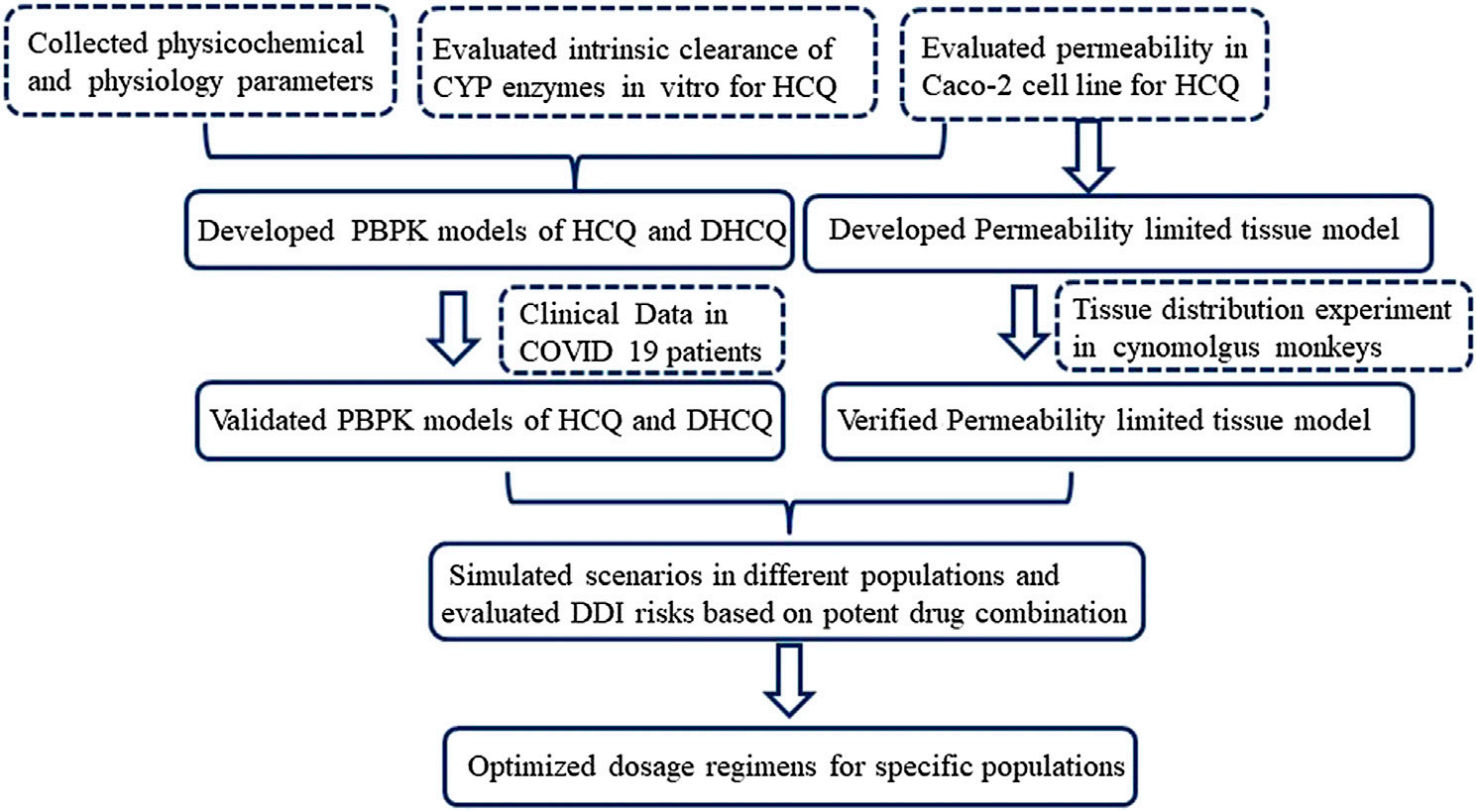
Strategy diagram of updating HCQ PBPK model and applying the model to support HCQ dose regimens in specific populations.

Firstly, our earlier HCQ PBPK model (Yao et al., 2020) was modified by applying a permeability-limited lung model to describe the dynamics of HCQ distribution into the lung tissue. Permeability parameters describing the diffusion of HCQ into small intestinal epithelial cells and lung cells were obtained through Caco-2 cells permeability experiments. Due to lack of HCQ concentration data in human lung tissue, we assumed that tissue distribution dynamics in human was similar to that in cynomolgus monkeys. The lung/plasma partition ratio (Kp) in the cynomolgus monkey was obtained from *in vivo* experiments (unpublished data, Institutional Animal Care and Use Committee (IACUC) No.16-120). Secondly, based on the structural formula of HCQ, we applied ADMET Predictor software (version 9.5, Simulationsplus, United States) to identify potential CYP metabolic enzymes of HCQ. These enzymes were also reported to be responsible for HCQ metabolism (Tanaka et al., 2004). The intrinsic clearance (CL_int_) values of major CYP isoforms, predicted from ADMET predictor, were measured using recombinant CYP isozymes. Then the contribution of each CYP isoform to HCQ’s overall metabolism was calculated according to these results. Thirdly, the PBPK model of DHCQ was established by collecting *in vitro* and *in vivo* data from the literatures (Tett et al., 1988; Tett et al.,1989; McLachlan et al., 1993; Somer et al., 2000; Xu et al., 2016; Al-Rawi et al., 2018; Collins et al., 2018; Balevic et al., 2019). To date, DHCQ is known to be associated with activity in treating rheumatoid arthritis (Munster et al., 2002; Chhonker et al., 2018). Its activity against SARS-CoV-2 was not obvious based on limited *in vitro* testing. Nonetheless, coupling HCQ model with DHCQ model within the same PBPK framework allows simultaneous evaluation of the exposure response relationships of both species, when needed. Fourthly, drug interaction potential of HCQ was considered in the model, including results of inhibition of OATP1A2 *in vitro* (Xu et al., 2016). Fifthly, the coupled HCQ-DHCQ PBPK mode was validated with regard to the prediction of blood and plasma concentration-time profiles or PK parameters from multiple literature reports (Supplementary Table S1). Moreover, we also compared model predictions to blood HCQ concentrations collected from COVID-19 patients who were treated with hydroxychloroquine sulfate in two recent clinical studies (Chinese Clinical Trial Registry Number: ChiCTR2000029898, ChiCTR2000029899; Supplementary Table S2). Finally, the updated and validated model was used to simulate PK characteristics of HCQ in specific populations (elderly, liver impairment, renal impairment, pregnant women and children) to support safe dosing regimens. Figure 1 displays the strategy diagram and Table 1 shows the simulation scenarios.

**TABLE 1:** Summary of the simulation scenarios in this manuscript.

(I) Simulation scenarios for specific populations with dosage regimen A			
Caucasian healthy population (20–50 years)			
Chinese healthy population (20–50 years)			
Sim-cirrhosis CP-A populations (20–50 and 65–70 years)			
Sim-cirrhosis CP-B populations (20–50 and 65–70 years)			
Sim-cirrhosis CP-C populations (20–50 and 65–70 years)			
Sim-renal-GFR-30–60 populations (20–50, 65–75 and 75–85 years)			
Sim-renal-GFR-less-30 populations (20–50, 65–75 and 75–85 years)			
Paediatric populations (0–0.5, 0.5–2, 2–6, 6–12, 12–17 years)			
Pregnant population (13, 25, 37 gestational weeks)			
Geriatric populations (65–75, 75–85, and 85–95 years)			
**(II)** **Simulation scenarios of potential drug drug interactions**			
Inhibitor	Treatment (days)	Dose regimen	HCQ dosage regimen (treatment)
Gemfibrozil (strong CYP2C8 inhibitor)	12	600 mg BID	Regimen A (D 3 - D 7)
Quinidine (strong CYP2D6 inhibitor)	12	200 mg QD	Regimen A (D 3 - D 7)
ritonavir (strong CYP3A4 inhibitor and weak CYP2D6 inhibitor)	12	100 mg QD	Regimen A (D 3 - D 7)

### Updating Hydroxychloroquine Physiologically-Based Pharmacokinetic Model

To evaluate the factors that may influence HCQ oral absorption, we updated the previous HCQ PBPK model using advanced dissolution, absorption and metabolism (ADAM) model in SymCYP Simulator (Version 18, Certara, United Kingdom), and conducted Caco-2 cell permeability study to obtain the apparent permeability coefficients (P_app_) of HCQ. Full-PBPK model was used to describe the distribution characteristics of HCQ. Elimination of HCQ in PBPK model was described using enzyme kinetics and renal clearance (CL_R_) in SimCYP. The recombinant isozymes informed by ADMET Predictor and reference (Tanaka et al., 2004), including CYP2C8, CYP2D6 and CYP3A4, were incubated with HCQ to determine the intrinsic clearance (CL_int_), respectively. The contribution of each CYP isoform to the overall hepatic metabolism was calculated according to the content of the specific CYP450 isoform in human liver. In order to quantify the accumulation of HCQ in lung tissue, we conducted tissue distribution study in cynomolgus monkeys to determine the Kp of lung/plasma (submitted as another manuscript, IACUC No.16-120), which was then used to inform permeability-limited lung model in human. Based on permeability limited kinetics and the characteristics of drug accumulation in lung tissue, permeability limited lung model was established. Parameters such as physicochemical parameters, protein binding, B/P ratio, and renal clearance for HCQ remain unchanged from previous model (Yao et al., 2020). Model parameters are summarized in Supplementary Table S3 in supplemental file.

### Development of Desethylhydroxychloroquine Model

*In vitro* study results showed that HCQ could be metabolized to DHCQ by CYP2C8, CYP2D6 and CYP3A4 (submitted as another manuscript). Therefore, DHCQ model was coupled to HCQ model (through CYP3A4, CYP2C8 and CYP2D6) in the liver. The physicochemical parameters of DHCQ were predicted by ADMET Predictor (Version 9.5, Simulationsplus, United States). The blood to plasma partition (B/P) ratio of DHCQ is predicted by the Prediction Toolbox in Simcyp software (Version 18, Certara, United Kingdom). Full PBPK model was also used to describe the distribution of DHCQ. Renal clearance of DHCQ was obtained from published data (Miller et al., 1991), and the liver microsomal clearance of DHCQ was fitted by one concentration-time profile of DHCQ in literature (Murphy et al., 1987). Model parameters are summarized in Supplementary Table S3 in supplemental file.

### Model Validation

The data for model validation were retrieved from the Pubmed and Embase databases with the keywords “hydroxychloroquine and clinical pharmacokinetics” and “desethylhydroxychloroquine”, and the retrieval time was from February 1955 to February 2020. Inclusion criteria for model validation are: 1) the study drug is hydroxychloroquine sulfate or hydroxychloroquine; 2) human as the research subjects had participated in clinical trials; 3) the literature reported the plasma and/or whole blood concentration-time profiles of HCQ or DHCQ. Exclusion criteria are: 1) when the dose and the characteristics of the population are similar, the concentration of the drug in whole blood or plasma is reported to be significantly higher or lower five times than the observed value of other similar studies; 2) when literature did not clearly describe the dosage and the study demographics; 3) literatures reported serum drug concentration. For the concentration-time profile figures in the references, the concentration data were obtained by Plot Digitizer (Version 2.26, GetData, China). For references that did not directly report PK parameters, the main PK parameters (area under the curve (AUC) and maximum concentration (C_max_) were calculated based on measured graphical data using Phoenix (Version 8.6, Certara, United Kingdom). The dose regimen and population characteristics from literatures and COVID-19 patients used in model validation are summarized in Supplementary Tables S1 and S2 in supplemental file.

The predictive performance of HCQ PBPK model was evaluated by comparing the predicted values with the observed values. A total of 11 clinical studies were used to validate the HCQ-DHCQ PBPK model. The trial design in Simcyp simulator was set to match population demographics (including ethnicity, age, and sex), as well as the dose regimens and blood collection time points of each reference report. Each simulation includes 10 trials with 10 subjects (n = 100). Evaluation criteria of HCQ PBPK predictive performance are: 1) the observed concentrations are within the 90% confidence interval (CI) of the predicted concentrations; 2) the ratios of simulated AUC and C_max_ values to that observed values are within a predefined boundary of 0.5 to 2.0-fold. In addition, comparing observed Kp of HCQ in cynomolgus monkeys with that predicted value in human was applied to support HCQ PBPK model predicting lung tissue drug concentrations in humans.

### Simulation in Specific Populations (Intrinsic Factors)

The dose regimen of HCQ (a loading dose of 600 mg twice daily of hydroxychloroquine sulfate given orally, followed by a maintenance dose of 200 mg given twice daily for 4 days orally, referred to as Regimen A in this paper) in the treatment of COVID-19 patients was used in two recent clinical studies (Chinese Clinical Trial Registry Number: ChiCTR2000029898, ChiCTR2000029899). Using the Regimen A, the plasma concentrations of HCQ in specific populations were simulated using the HCQ-DHCQ PBPK model.

Unless otherwise specified, physiological models of these populations (virtual populations that are drug independent) within in SimCYP simulator database were directly used without modification. These populations included Caucasian healthy volunteers (baseline population, 20–50 years old), geriatrics, cirrhotic patients with Child-Pugh scores (CP) A, B, and C (mild, moderate and severe hepatic impairment, respectively), renal impairment with glomerular filtration rate (GFR) 30–60 ml/min (moderate renal impairment) and less 30 ml/min (severe renal impairment), pregnant women, and pediatrics. According to the physiology characteristics, geriatric population was divided into subsets of 65–75, 75–85, and 85–95 years old. We also simulated HCQ PK in more specific geriatric populations with liver or kidney impairment and geriatric populations taking strong CYP2C8, CYP2D6 and CYP3A4 modulators (See *DDI Simulations* below). Due to the age limitation of virtual populations of liver and renal dysfunction in SimCYP, we attempted such geriatric simulations in liver impairment populations with narrow age band of 65–70 years old, and renal impairment populations of with the age 65–75 and 75–85 years old. To simulate HCQ in pregnant women, we selected gestational weeks 13, 25, and 37 to represent first, second and third trimesters. Pediatric population was divided into six groups, including 0–1 month, 1–6 months, 0.5–2, 2–6, 6–12, and 12–17 years old. For other populations, the age range was set as 20–50 years old. Except for pregnant women, the ratio of male to female was set at 1:1. Each simulation includes 10 trials with 10 subjects (n = 100). Comparison of HCQ exposure of a specific population to that of baseline population (Healthy Caucasians of 20–50 years old, male:female = 1:1) using Regimen A was performed.

### DDI Simulations

This work focuses on predicting the effects of concomitant medications on the PK of HCQ. In order to understand the worst case scenarios of potential drug-drug interactions (DDI) on the plasma exposure of HCQ, the baseline treatment regimen of HCQ (Regimen A) was used in all DDI simulations and DDI potential was evaluated using baseline population (Healthy Caucasians of 20–50 years old, male:female = 1:1) taking Regimen A as reference (see *Simulation in Specific Populations (Intrinsic Factors)* above). We focused on the effects of CYP2C8, CYP2D6 and CYP3A4 perpetrators on the exposure of HCQ because they are the main CYP isoforms based on *in vitro* experiments presented in our work and reference report (Tanaka et al., 2004). DDI simulations included gemfibrozil (strong CYP2C8 inhibitor), quinidine (strong CYP2D6 inhibitor) and ritonavir (strong CYP3A4 inhibitor and weak CYP2D6 inhibitor). The default drug models and default dose regimens of these perpetrators in SimCYP were used for all DDI simulations. Besides baseline population, DDI simulations were conducted in geriatric sub-populations mentioned above. The design of drug interaction simulation scenarios can be found in Table 1.

## Results

### Physiologically-Based Pharmacokinetic Model Development

**FIGURE 2:**
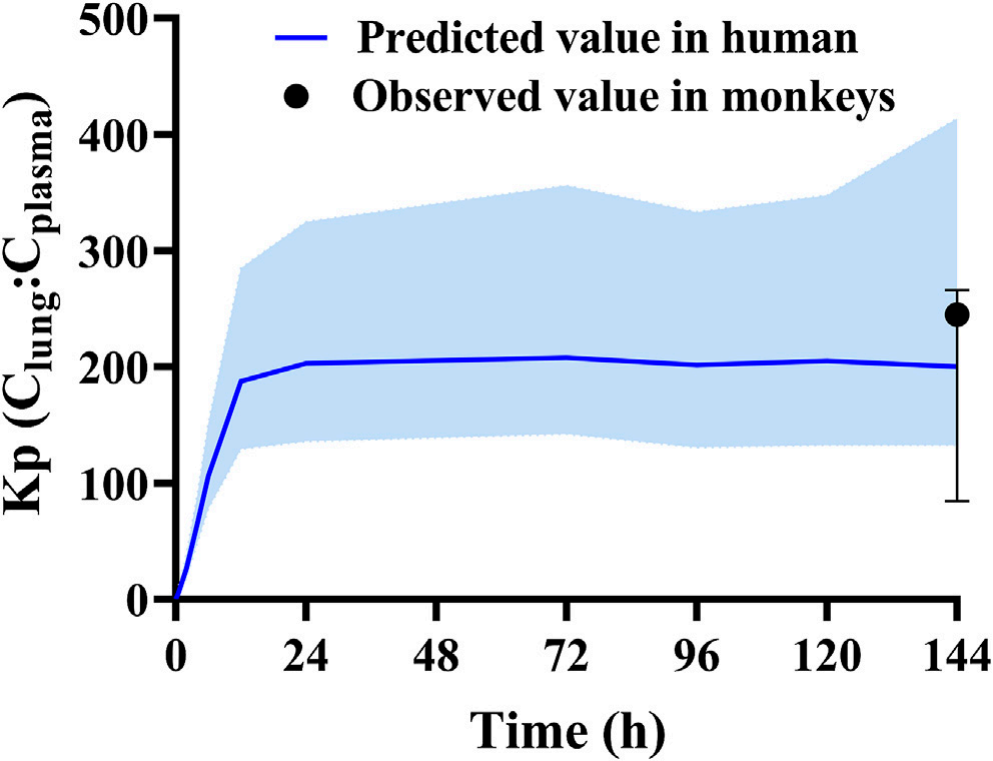
Validated results of permeability limited lung model with observed Kp values (plasma concentration vs. lung concentration) in monkeys and predicted Kp values in human.

The final HCQ and DHCQ model parameters are shown in Supplementary Table S3 in supplemental file. Of note, several parameters are obtained by conducting *in vitro* experiments. Caco-2 cell monolayer study is conducted to obtain P_app_ (3.68 × 10^–6^ cm/s). Based on in silico prediction, reference report (Tanaka et al., 2004), *in vitro* study using recombinant CYP isoforms and human liver microsome co-incubation with specific inhibitors, the main metabolic enzymes are determined to be CYP2C8, CYP2D6 and CYP3A4. Except for renal clearance, CYP2C8, CYP2D6 and CYP3A4 contribute to 37.3, 19.3, and 16.7% of total clearance in the HCQ-DHCQ PBPK model, respectively. We also conducted pharmacokinetic study in monkeys to evaluate the accumulation of HCQ in lung tissue. Kp of lung concentration to plasma concentration at trough concentration on day 6 is around 200, which can be used for informing permeability-limited lung model (shown in Figure 2). Inhibition of OATP1A2 *in vitro* is collected from literature (Xu et al., 2016). HCQ can be excreted by kidney, which account for 16–30% of total clearance (Miller et al., 1991; Fox, 1993). These absorption, metabolism, permeability limited lung model, as well as the development of PBPK model of major HCQ metabolite, DHCQ, are integrated into our previously published HCQ PBPK model (Yao et al., 2020).

### Physiologically-Based Pharmacokinetic Model Validation

**FIGURE 3:**
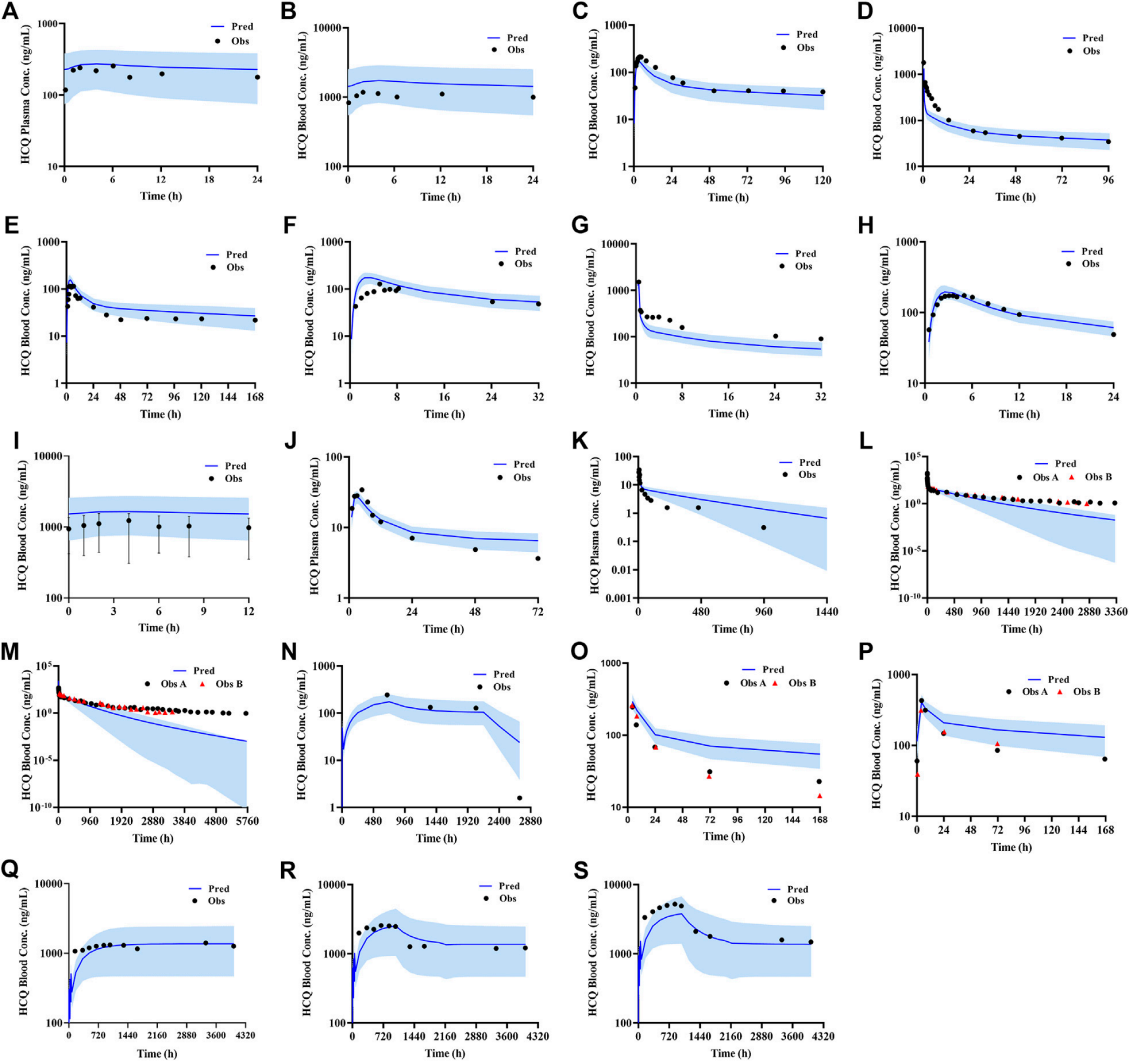
Validated results of HCQ PBPK model with the dosage regimens and sampling times listing in Supplementary Table S1.

**FIGURE 4:**
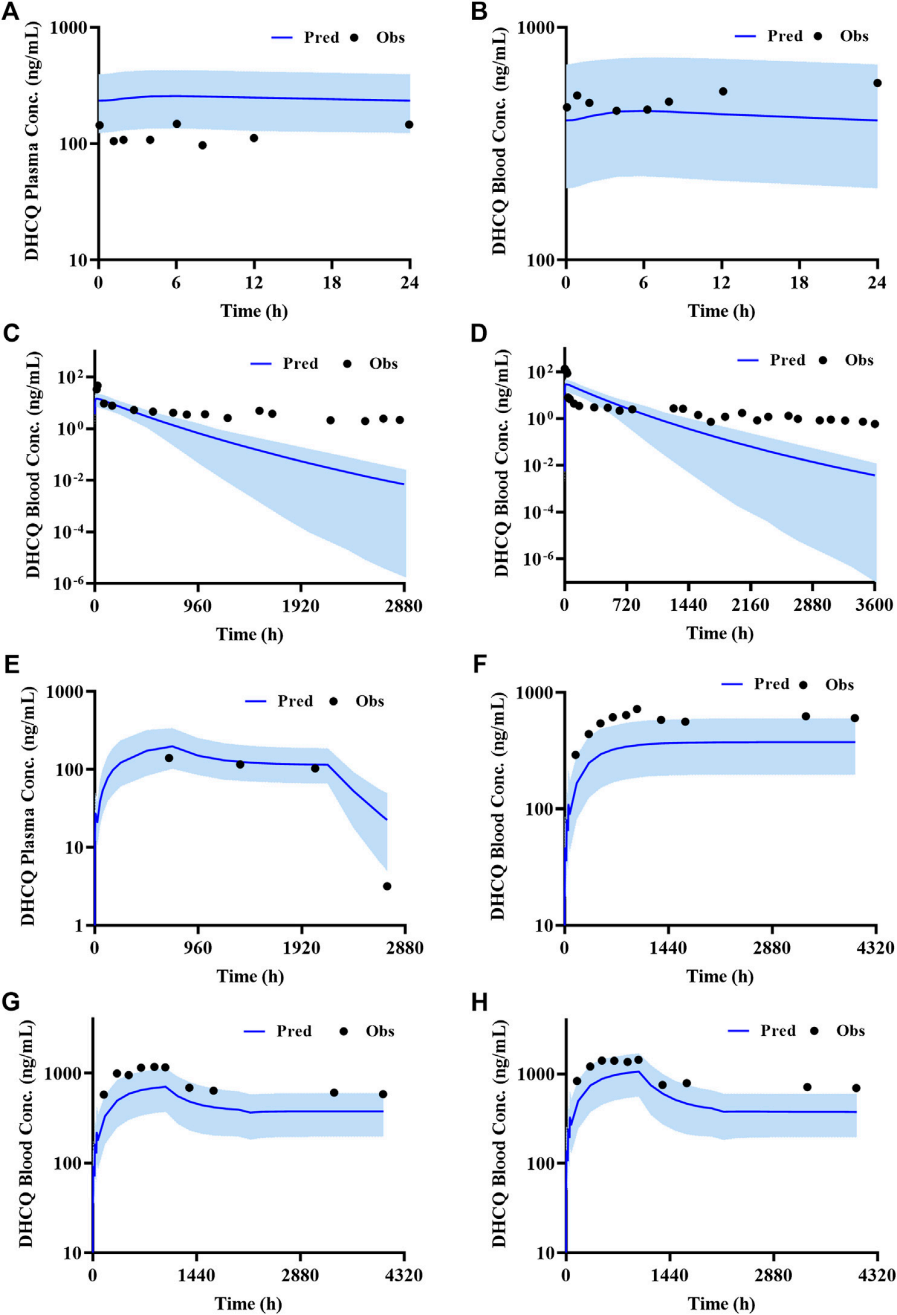
Validated results of DHCQ PBPK model with the dosage regimens and sampling times listing in Supplementary Table S1.

**FIGURE 5:**
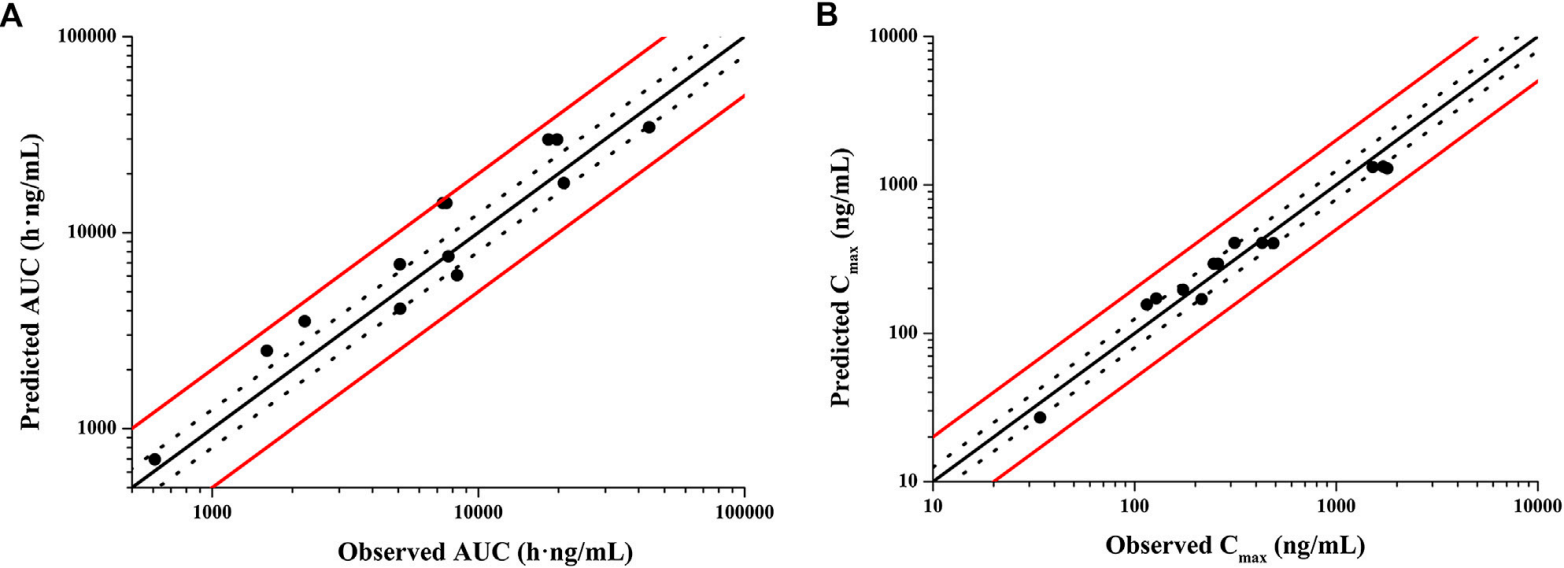
Validation of HCQ PBPK model by comparing model predicted and observed PK parameters **(A)**: AUC; **(B)**: C_max_. Red solid lines are predefined 2-fold boundary; black dotted lines are predefined 1.25-fold boundary.

**FIGURE 6:**
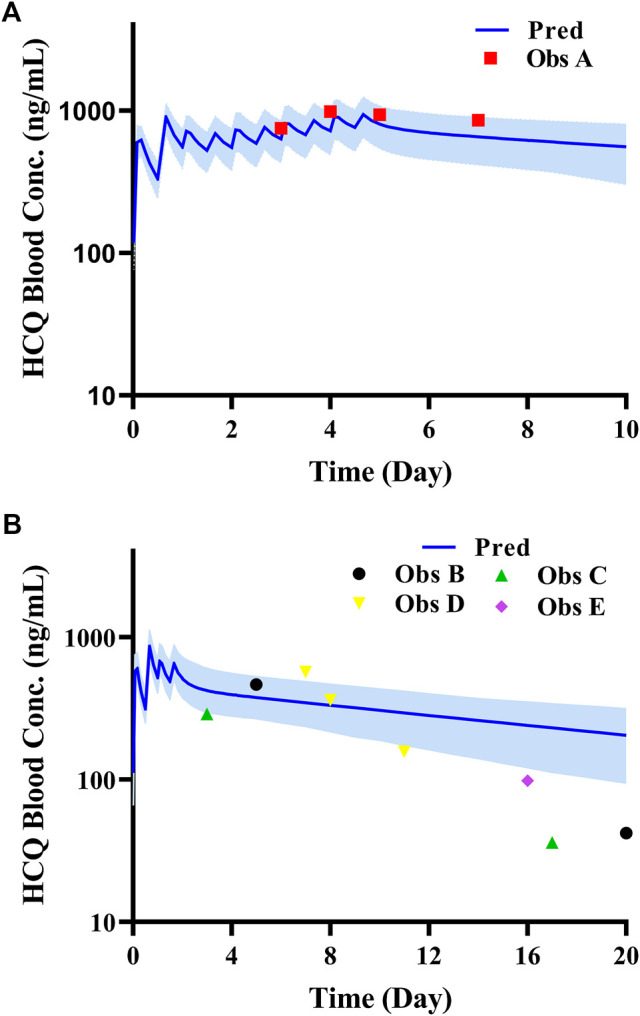
Comparison of HCQ PBPK model with PK data in COVID-19 patients under different dosing regimens (Data source: ChiCTR2000029898, ChiCTR2000029899). **(A)**: Patient with dosage regimen: D1 600 mg BID, D2-5 200 mg BID; **(B)**: four patients with dosage regimen: D1 600 mg BID, D2 200 mg BID.

According to exclusion criteria mentioned above, we exclude six papers (Iredale et al., 1993; McLachlan et al., 1993; Lim et al., 2009; Costedoat-Chalumeau et al., 2013; Charlier et al., 2018; Balevic et al., 2019) as HCQ-DHCQ PBPK model validated data. A total of nineteen HCQ and eight DHCQ blood and/or plasma concentration-time profiles from an additional eleven literatures are used to validate the HCQ-DHCQ PBPK model. All the observed data are within 90% confidence interval (CI) of predicted results. Due to the deficiency of reported concentration data, PK parameters (AUC and C_max_) in six PK profiles from literatures cannot be calculated. For the rest, 13/13 of the ratios about HCQ-DHCQ PBPK model predicted HCQ AUC and C_max_ over observed values are within predefined boundary of 2-fold, 4/13 AUC ratios and 7/13 C_max_ ratios are within a narrower 1.25-fold. There are four papers containing DHCQ clinical data and eight PK profiles are collected. One of these profiles is used to develop the DHCQ model (Murphy et al., 1987) and seven profiles are used for validation. The prediction performance of DHCQ model is satisfactory with all 8/8 observed concentrations from four studies captured within the 90% CI of model predicted concentrations. The validated results of HCQ and DHCQ model based on eleven and four literatures can be found in Figure 3 and Figure 4, respectively. The visual presentation of AUC and C_max_ ratio are displayed in Figure 5 (The values of observed and predicted AUC and C_max_ ratio are showed in Supplementary Table S4). In addition, model simulations appear to capture sparse whole blood PK data from five COVID-19 patients recently treated with HCQ (Figure 6).

### Predicting Hydroxychloroquine PK in Specific Populations

Because most virtual specific populations in the SimCYP database are established based on Caucasians, the HCQ exposure in Caucasians healthy volunteers is used as a baseline to evaluate the exposure differences between specific populations and Caucasians healthy adults under the same dose regimen. Assuming the same plasma exposure of HCQ results in the same safety profiles of the drug among various populations, we used the HCQ-DHCQ PBPK model to simulate PK profiles for each specific population by comparing plasma C_max_ of HCQ in healthy adults (baseline population, healthy Caucasians 20–50 years old, male:female = 1:1) under Regimen A. As the trend of HCQ exposure variation from baseline population appears to be similar between plasma and lung tissue in different populations, herein, only the ratios of plasma concentration in special populations vs. baseline population are described in detail below, and lung tissue concentration of HCQ can be found in Figure 8.

**FIGURE 7:**
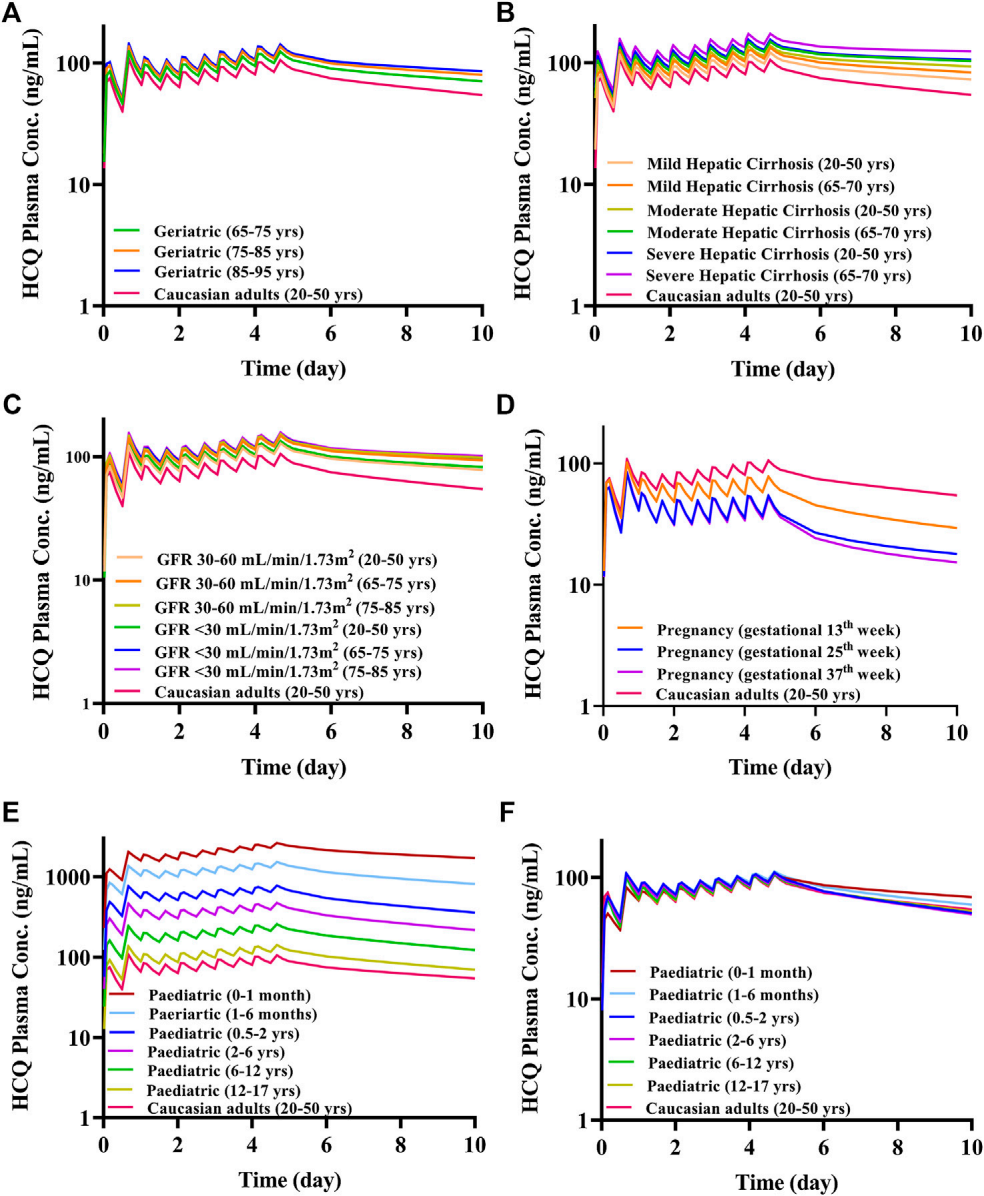
The mean plasma exposure of HCQ in specific populations with Regimen A (except for F). **(A)** Geriatrics; **(B)** liver impairment; **(C)** renal impairment; **(D)** pregnant women; **(E)** pediatric; **(F)** pediatric with body weight-adjusted dosage regimens.

**FIGURE 8:**
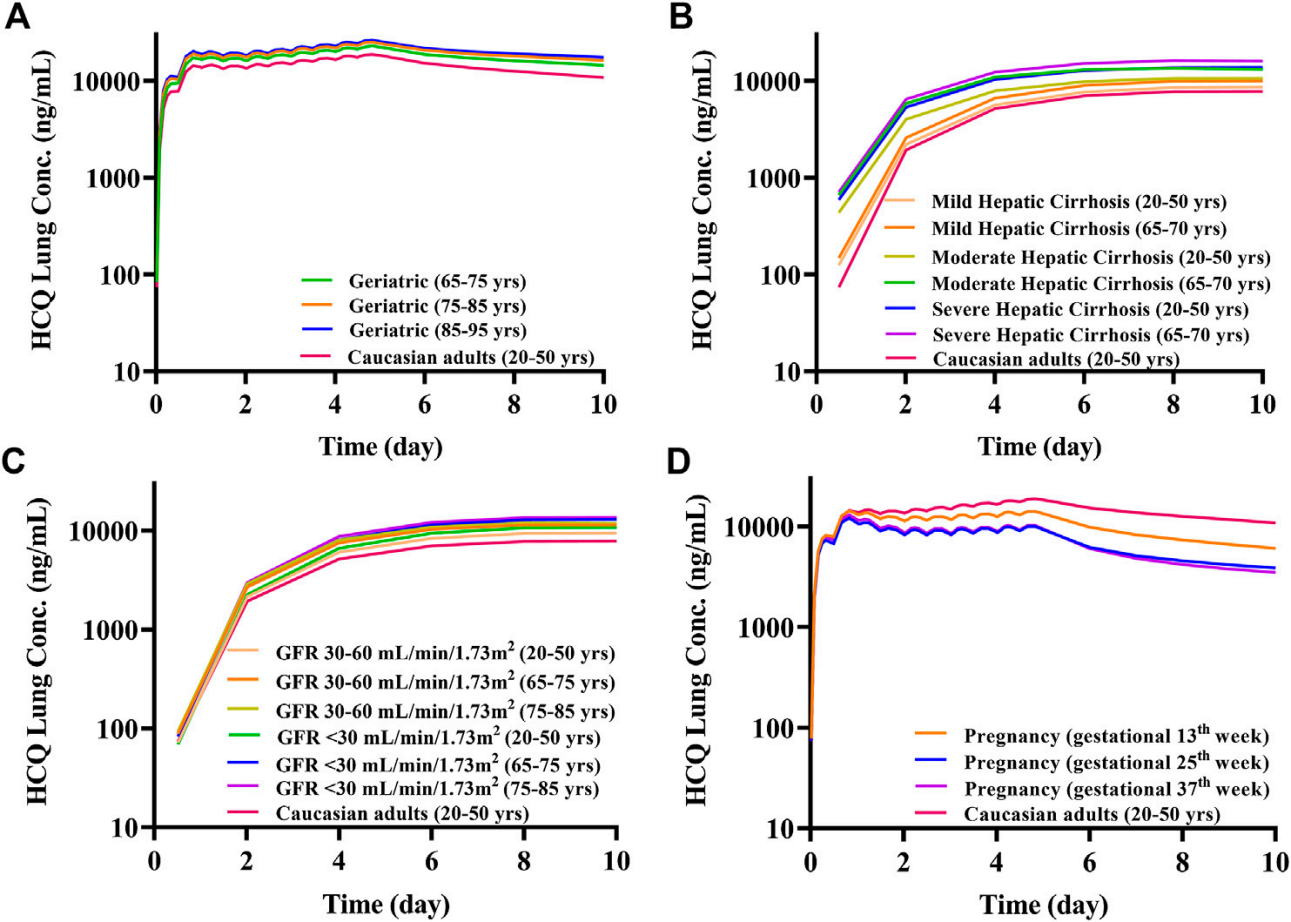
The mean lung tissue exposure of HCQ in specific populations with Regimen A. **(A)** Geriatrics; **(B)** liver impairment; **(C)** renal impairment; **(D)** pregnant women.

### Geriatrics and Organ Dysfunction

The geriatric population aged 65–95 years old had similar systemic exposure compared with healthy adults (baseline population), which are summarized in Figure 7A, Table 2 and Figure 8A. However, when the geriatric population has severe liver or renal impairment, simulated plasma HCQ values are increased.

**TABLE 2:** The mean ratio of predicted PK parameters of geriatrics at different age ranges to that of adults^a^.

	Geriatric (65–75 years)	Geriatric (75–85 years)	Geriatric (85–95 years)
R_Cmax_plasma_	1.17	1.29	1.35
R_AUC_plasma_	1.20	1.32	1.38
R_Cmax_lung_	1.22	1.33	1.40
R_AUC_lung_	1.24	1.36	1.44

a Adults: baseline population, 20–50 years old healthy Caucasians.

Compared with non-cirrhotic subjects, liver impairment patients with CP score A, B, and C (20–50 years old and 65–70 years old) have higher exposure, the ratios of main plasma PK parameters (C_max_, AUC) compared to baseline population (healthy subjects 20–50 years old) ranges from 1.15 to 1.77. Simulated HCQ exposure positively correlated with the degree of liver impairment and age, as patients have the highest C_max_ ratio (e.g., 65–70 years old with severe liver impairment C_max_ ratio is 1.64). Results are exhibited in Figure 7B, summarized in Table 3 and Figure 8B.

**TABLE 3:** The mean ratio of predicted PK parameters of liver impairment population at different age ranges to that of adults^a^.

	Cirrhosis-A (20–50 years)	Cirrhosis-B (20–50 years)	Cirrhosis-C (20–50 years)	Cirrhosis-A (65–70 years)	Cirrhosis-B (65–70 years)	Cirrhosis-C (65–70 years)
R_Cmax_plasma_	1.15	1.31	1.47	1.26	1.41	1.64
R_AUC_plasma_	1.18	1.39	1.56	1.31	1.51	1.77
R_Cmax_lung_	1.22	1.56	1.97	1.38	1.74	2.28
R_AUC_lung_	1.25	1.63	2.07	1.42	1.82	2.40

a Adults: baseline population, 20–50 years old healthy Caucasians.

According to the simulation results (Figure 7C; Table 4), the plasma exposure of patients with renal impairment (20–50 years old, 65–75 years old and 75–85 years old) is similar to that of adults, and the ratio of main plasma PK parameters (C_max_, AUC) to adult are in the range of 1.22–1.56. The exposure of HCQ in lung tissue is shown in Figure 8C.

**TABLE 4:** The mean ratio of predicted PK parameters of renal impairment population at different age ranges to that of adults^a^.

	Renal-GFR 30–60 (20–50 years)	Renal-GFR 30–60 (65–75 years)	Renal-GFR 30–60 (75–85 years)	Renal-GFR less 30 (20–50 years)	Renal-GFR less 30 (65–75 years)	Renal-GFR less 30 (75–85 years)
R_Cmax_plasma_	1.22	1.40	1.45	1.27	1.44	1.49
R_AUC_plasma_	1.26	1.46	1.51	1.32	1.51	1.56
R_Cmax_lung_	1.30	1.53	1.57	1.48	1.72	1.77
R_AUC_lung_	1.33	1.57	1.63	1.52	1.78	1.83

a Adults: baseline population, 20–50 years old healthy Caucasians.

### Pregnant Women

The exposure of HCQ in pregnant women at gestational weeks of 13, 25 and 37 weeks are simulated under Regimen A. The ratios of plasma C_max_ and AUC are within the range of 0.50–0.74 and 0.44–0.69 compared with baseline population (Figure 7D; Table 5). Figure 8D displays the exposure of HCQ in lung tissue in pregnant women.

**TABLE 5:** The mean ratio of predicted PK parameters of pregnant women at different gestational weeks to that of adult^a^.

	Pregnancy 13 weeks	Pregnancy 25 weeks	Pregnancy 37 weeks
R_Cmax_plasma_	0.74	0.51	0.50
R_AUC_plasma_	0.69	0.46	0.44
R_Cmax_lung_	0.76	0.54	0.55
R_AUC_lung_	0.73	0.51	0.52

a Adults: baseline population, 20–50 years old healthy Caucasians.

### Pediatrics

Under the Regimen A, the simulated plasma HCQ AUC and C_max_ are significantly increased as age decreases. The simulated PK profiles in pediatric are shown in Figure 7E. Dose adjustments based on body weight were explored using PBPK modeling in pediatric patients by matching systemic exposure in baseline population under Regimen A. In order to match plasma C_max_ after the first dose in baseline population under Regimen A (600 mg, assuming a 70 kg healthy adult), body weight based starting (loading) doses are: 5.4 mg/kg for infants less than 1 month, 6.0 mg/kg for infants aged 1–6 months, 6.3 mg/kg for infants aged 0.5–2 years old, 6.6 mg/kg for children aged 2–6 years old, 6.9 mg/kg for children aged 6–12 years old, and 6.9 mg/kg for adolescent aged 12–17 years old. Meanwhile, the suggested maintenance doses are approximately one third of the loading dose. After dose adjustment, the exposure in children is similar to adults and the results are shown in Figure 7F and Table 6. As permeability limited lung model can-not predict the exposure of HCQ in lung tissue in pediatric, only plasma exposure is shown in this paper.

**TABLE 6:** The mean ratio of predicted PK parameters of children at different age ranges to that of adults^a^.

Age of paediatric	0–1 month	1–6 months	0.5–2 years	2–6 years	6–12 years	12–17 years
Loading dose (mg/kg)	5.4	6.0	6.3	6.6	6.9	6.9
R_Cmax_plasma_	1.01	1.06	1.04	1.02	1.00	1.00
R_AUC_plasma_	1.08	1.09	1.04	1.02	0.98	1.00

a Adults: baseline population, 20–50 years old healthy Caucasians.

R: ratio between specific pediatric population taking a weight-based regimen and baseline population taking Regimen A. The suggested maintenance doses are one third of the loading dose (See *Results*). AUC: Area under the curve over 10 days of dosing.

### DDI Evaluation

In the presence of strong CYP2C8, CYP2D6 and CYP3A4 inhibitors, simulated HCQ exposure is not significantly affected in baseline healthy adult population (20–50 years old). The plasma AUC increases by about 9–19% (Table 7) when coadministration with inhibitors. However, plasma exposure increases by up to ∼56% in elderly subjects taking these strong inhibitors compared to baseline healthy adult population, respectively.

**TABLE 7:** The mean ratio of predicted PK parameters for subjects with combination of HCQ and CYP2C8/CYP2D6/CYP3A4 inhibitor to that for subjects* with HCQ alone.

Gemfibrozil (CYP2C8 strong inhibitor) + HCQ				
	20–50 years	65–75 years	75–85 years	85–95 years
R_Cmax_plasma_	1.15	1.32	1.45	1.50
R_AUC_plasma_	1.19	1.38	1.51	1.56
R_Cmax_lung_	1.15	1.37	1.50	1.55
R_AUC_lung_	1.17	1.41	1.55	1.60

Quinidine (CYP2D6 strong inhibitor) + HCQ				
	20–50 years	65–75 years	75–85 years	85–95 years
R_Cmax_plasma_	1.08	1.27	1.38	1.46
R_AUC_plasma_	1.09	1.31	1.42	1.49
R_Cmax_lung_	1.07	1.32	1.42	1.50
R_AUC_lung_	1.08	1.35	1.45	1.23

Ritonavir (CYP3A4 strhong inhibitor and CYP2D6 weak inhibitor) + HCQ				
	20–50 years	65–75 years	75–85 years	85–95 years
R_Cmax_plasma_	1.11	1.29	1.42	1.49
R_AUC_plasma_	1.12	1.34	1.46	1.53
R_Cmax_lung_	1.10	1.34	1.45	1.53
R_AUC_lung_	1.11	1.37	1.49	1.57

subjects*: baseline population, 20–50 years old healthy Caucasians.

R: ratio between subjects taking HCQ with a CYP2C8/CYP2D6/CYP3A inhibitor and subjects taking HCQ only under Regimen A. AUC: Area under the curve over 10 days of dosing.

## Discussion

The HCQ-DHCQ PBPK model with detailed mechanisms of HCQ liver metabolism and lung distribution was developed and validated using observed human PK data. The in-depth lung tissue distribution of the model was informed by lung tissue and plasma data obtained in monkeys. This model was used to predict PK characteristics of HCQ in specific populations and drug interaction between HCQ and CYP2C8/CYP2D6/CYP3A4 perpetrators. In addition, the HCQ-DHCQ PBPK model included a more mechanistic oral absorption model (ADAM model) is coupled with a PBPK model of major metabolite DHCQ (Miller et al., 1991). These additional features can help us further studying clinical pharmacology questions related to HCQ oral absorption process and safety effects of DHCQ in patients taking hydroxychloroquine sulfate for both approved indications and for COVID-19 in investigational trials. To our knowledge, this is the first report about updating key absorption, metabolism and distribution mechanisms of HCQ PBPK model through the conduct of nonclinical experiments. The manuscript also compares PBPK model prediction with sparse HCQ PK data in whole blood from limited COVID-19 patients.

Unless otherwise specified (e.g., pediatric populations), Regimen A was used to simulate HCQ PK in all specific populations. Because dosing in Regimen A is similar to that recommended in HCQ product label, exposure under regimen A in healthy subjects (20–50 years old) serves as reference exposure for safety. Indeed, Regimen A was used in COVID-19 patients in our recent clinical studies and the drug was generally well tolerated in these patients (ChiCTR2000029898, ChiCTR2000029899, unpublished data). HCQ is known to have potential risks such as hypoglycemia, cardiac interstitial prolongation of QTc, and eye discomfort. Historically, these events remain rare and are mainly found in patients who were overdosed or after chronic uses (Chen et al., 2006; de Olano et al., 2019).

Based on *in vitro* results and contribution of renal clearance (∼26.7%), we report here that CYP2C8, CYP2D6, and CYP3A4 contribute to 37.3, 19.3, and 16.7% of total clearance of HCQ. This suggests that, even for CYP2C8 which has the highest contribution of HCQ metabolism, exposure of HCQ in the presence of a strong CYP2C8 inhibitor is unlikely to drastically increase due to existed multiple elimination ways and the larger K_i_ of gemfibrozil. Indeed, DDI simulations suggested marginal increase in HCQ exposure by coadministration with strong CYP isozyme inhibitors in non-elderly subjects (Table 7, 20–50 years old group).

Although people of all ages can be infected by SARS-CoV-2, the conversion rate to critical illness might be higher in the elderly population with underlying comorbidities. The plasma exposure of HCQ may markedly increase in certain elderly subpopulations who may have decreased liver and/or kidney functions, meanwhile taking other medications that can inhibit CYP2C8, CYP2D6 or CYP3A4 isozyme. Therefore, HCQ dosage regimen should be carefully determined to alleviate adverse events. For example, in order to match systemic exposure to that in baseline population taking Regimen A, a 50% dose reduction might need to be considered in elderly taking strong CYP2C8/CYP2D6 inhibitor (85–95 years old, Table 7) or having moderate and severe liver impairment (65–70 years old, Table 3). Similarly, it appears that a weight-based dosing may be needed in young children to match plasma exposure of HCQ to that in baseline population taking Regimen A (Table 6). The exposure-matching strategy using PBPK modeling was recently reported by Verscheijden et al. to propose safe doses of chloroquine for pediatric patients who may need to take the drug to treat COVID-19 (Verscheijden et al., 2020). Updating our model with detailed contributions of major metabolizing enzymes enabled the use of know ontogeny in PBPK framework for proper prediction of HCQ exposure in children. Conversely, both simulated HCQ plasma and lung exposure can decrease in pregnant women (Table 5). Decreased drug exposure may warrant dose increase in these specific populations to ensure adequate drug levels in target organ, without exceeding safety exposure in plasma.

For all these specific populations, little or no clinical data exist to allow validation of our simulations. Indeed, one clinical study reported HCQ exposure in pregnant women with rheumatoid arthritis (Balevic et al., 2019). While, we decided not to use this data for validating our model because i) the study reported serum concentrations which may be contaminated by drug released from red blood cells during sample preparation (see inclusion and exclusion criteria of model verification); ii) proinflammatory factors such as interleukin-6 can down-regulate the activity of liver metabolism enzymes such as CYP3A (Machavaram et al., 2013), which may not compensate the up-regulation of CYP3A activity during pregnancy. With regard to simulation of HCQ PK in COVID-19 patients (Figure 6), we used population model representing healthy subjects. This may not be adequate because COVID-19 patients may have underlying pathophysiological changes that may influence the PK of HCQ. Therefore, all specific population predictions presented in this manuscript eventually will be verified by clinical studies. Nonetheless, the HCQ-DHCQ PBPK model allows mechanistic assessment of HCQ PK in these populations and contributes optimal dose selection of untested clinical scenarios. This is especially important under pandemics of an emerging but life-threatening pathogen.

Regarding DDI assessment, we concentrated in this report the effect of CYP modulators on the PK of HCQ. Some investigations indicated that HCQ could block OAT1, OAT2, OAT4, OATP1B1, OATP1B3, OATP2B1, OCT2, OCTN1 slightly and had no effect on OAT3, OCT1, OCT3 and OCTN2(Xu et al., 2016). Potential DDI may exist between HCQ and digoxin (Leden, 1982), likely due to HCQ’s effect on P-glycoprotein. Recent reports suggested that HCQ should be combined with azithromycin to treat COVID-19 (Gautret et al., 2020a; Gautret et al., 2020b). To our knowledge, azithromycin is not a CYP2C8/CYP2D6/CYP3A4 inhibitor therefore unlikely will affect the PK of HCQ. However, it is not clear whether HCQ can influence the PK of azithromycin. Future study is warranted to determine DDI liability of HCQ as a perpetrator of enzymes and transporters.

Although the HCQ-DHCQ PBPK model has been improved significantly from the original model (Yao et al., 2020), several limitations warrant further research to fill the knowledge gaps (such as the effect of disease states on HCQ PK), and we hereby focus on assumptions related to the update of HCQ’s lung model. Passive permeability of drug in the lung compartments was obtained from the result of Caco-2 cell lines. The relationship between Caco-2 and Calu-3 cell lines (the preferred experiment *in vitro* for evaluating passive permeability in lung tissue), is obtained from an equation ($\text{Calu}-3P_{\text{app}}\left[10^{-7}\text{cm}/\text{s}\right]=0.5881\times \text{Caco}-2\;P_{\text{app}}\left[10^{-7}\text{cm}/\text{s}\right]+4.5594;r^{2}=0.76$) described in literature (Gaohua et al., 2015). To better describe the characteristics of HCQ accumulation in lung tissue, we applied an apparent active transport mechanism (CL_int,T_, Supplementary Table S3) in the lung model to describe the accumulation of HCQ distributed into the lung tissue over time. Here we assumed that distribution characteristics are the same between human and animals. Although the model seems to capture single time point of Kp observed in cynomolgus monkeys (Figure 2), this assumption still requires confirmation ultimately using human data. In addition, based on pre-defined criteria (2.0-fold, 1.25-fold and 90% CI), not all reported PK profiles were well described by PBPK simulations, especially for sparse samples collected in five COVID-19 patients taking HCQ. We consider that there are four main reasons causing such discrepancies: i) data obtaining through digitization may introduce error; ii) some of the studies have a small sample sizes to inadequately represent PK behaviors of subjects being studied in them (e.g., Figure 6B); iii) we assumed patients infected by SARS-Cov-2 can be adequately represented by virtual healthy volunteer population in the model. The disease effect on the PK of HCQ has not been elucidated; and iv) although we exclude studies reporting serum concentrations, we do not know if all studies included in our analysis had plasma samples carefully prepared without breaking red blood cells, as HCQ has a higher B/P ratio. Lastly, DHCQ was formed by CYP2C8, CYP2D6 and CYP3A4, however the overall fraction of HCQ metabolized to DHCQ is still unclear. We will continue conducting more experiments *in vitro* and collecting more data to validate the model.

As the understanding of HCQ pharmacology and COVID-19 disease progression expands, mechanisms other than direct antiviral effect may contribute to potential clinical benefit of HCQ in COVID-19 patients. Although the simulated free HCQ plasma concentration was much lower than its anti-SASR-CoV-2 EC_50_ *in vitro*, the HCQ concentration in lung tissue was comparable to intracellular EC_50_ (unpublished data) due to its highly intracellular accumulation characteristics. Indeed, Jansson-Löfmark et al. (Jansson-Löfmark et al., 2020) have reported that EC_50_ is not a indicator to evaluate the efficacy of drug *in vivo*, as many factors that can influence in vitro-in vivo extrapolation, as illustrated by Jansson-Löfmark et al. Therefore, the efficacy of HCQ as an anti-SARS-CoV-2 agent may not be estimated merely based on *in vitro* data. Because of the difference between tissue concentration and plasma concentration, dose regimen design of HCQ should consider the plasma concentration as well as lung tissue concentration (Rowland Yeo et al., 2020; Yao et al., 2020). Other evidence, especially clinical findings, should also be integral part of the dose selection process (Yao et al., 2020; Zhang et al., 2020). Although the HCQ-DHCQ PBPK model can simulate other tissue’s concentrations, these predictions remain to be validated. Despite above limitations and considerations, our HCQ-DHCQ PBPK model provides a mechanism-based framework for clinicians that can be readily used to simulate plasma and lung PK profiles under different dose regimens.

## Conclusion

In this paper, we updated HCQ PBPK model by integrating its metabolism and distribution mechanism, and simulated HCQ pharmacokinetic characteristics in specific populations with underlying intrinsic and extrinsic characteristics. Although these simulated scenarios have not been verified by clinical data, the HCQ-DHCQ PBPK model can effectively provide supports in dosing recommendations in specific patient populations taking HCQ for both approved indications and for combating COVID-19.

## Data Availability

The raw data supporting the conclusions of this article will be made available by the authors, without undue reservation.
